# Role of Surgery in Management of Osteo-Articular Tuberculosis of the Foot and Ankle

**DOI:** 10.2174/1874325001711010633

**Published:** 2017-07-31

**Authors:** Mandeep Singh Dhillon, Vikas Agashe, Sampat Dumbre Patil

**Affiliations:** 1 Deptt of Orthopaedics, Postgraduate Institute Of Medical Education & Research, Chandigarh, India; 2 Visiting Consultant in Orthopaedics, P.D. Hinduja Hospital & Research centre, Kohinoor Hospital, Mumbai, India; 3 Director and Head of Orthopedics, Noble Hospital, Pune, Maharashtra, India

**Keywords:** Ankle arthrodesis, Foot infection, Hindfoot fusion, Tuberculosis Foot Ankle, Midfoot Tuberculosis, foot reconstruction

## Abstract

**Background:**

Tuberculosis of the foot and ankle still remains to be a significant problem, especially in the developing countries, and with an increase in incidence in immunosuppressed patients. Treatment is mainly medical using multidrug chemotherapy; surgical interventions range from biopsy, synovectomy and debridement, to joint preserving procedures like distraction in early cases, and arthrodesis of hindfoot joints and the ankle in advanced disease with joint destruction.

Surgical Options: All procedures should be done after initiating appropriate medical management. The ankle is the commonest joint needing intervention, followed by the subtalar and talo-navicular joint. Forefoot TB limited to the bone rarely needs surgical intervention except when the infective focus is threatening to invade a joint. Articular disease can spread rapidly, so early diagnosis and treatment can influence the outcome. Surgical interventions may need to be modified in the presence of sinuses and active disease; fusions need compression, and implants have to be chosen wisely. External fixators are the commonest devices used for compression in active disease, but intramedullary nails better stabilize pantalar arthrodesis. Arthroscopy has become a valuable tool for visualizing the ankle and hindfoot joints, and is an excellent adjunct for arthrodesis by minimally invasive methods.

**Conclusion:**

Although Osteoarticular Tb involving the foot and ankle is largely managed with chemotherapy, specific indications for surgical intervention exist. Timely done procedures could limit joint destruction, or prevent spread to adjacent joints. Fusions are the commonest procedure for sequelae of disease or for correcting residual deformity.

## INTRODUCTION & EPIDEMIOLOGY

Osteo-articular tuberculosis, although still uncommon, has shown a resurgence in recent times due to the advent of immune compromised patients and a geriatric shift in the population profile; western countries are seeing cases from migrant groups and drug addicts [1-3]. In the developing world, the incidence is still high, although less than 2% of the cases involve the foot and ankle [4-6]. Diagnostic delays are frequent due to its rarity in the footand lack of awareness, and inadequate management protocols lead to suboptimal results. The mainstay treatment remains appropriate multi-drug regimen antibiotic, but some situations demand surgical interventions. Tuberculosis of the foot maybe classified as synovial (rare), osseous (frequent), or articular (indicative of late stage) (Figs. **1a**, **b**, **c**). The disease may spread across the whole hindfoot, and we often encountered multifocal involvement in the same foot. Isolated bony involvement is uncommon, and the presentation in children (Figs. **2a**, **b**) and adults may be quite different (Figs. **3a**, **b**, **c**). All diagnostic modalities like CT and MRI should supplement routine X-rays when there is clinical suspicion and elevated ESR, in patients with unusual foot pain.

Multi drug anti-tubercular chemotherapy cure most of the cases in early stage, with near complete resolution of disease. Operative intervention in the foot is only indicated for non-responsive cases, uncertain diagnosis, for preventing joint involvement when a juxta-articular focus threatens to invade a joint, or for a painful joint with an unacceptable deformity after resolution of the disease. In many occasions the disease is noted in two adjacent bones [kissing lesions]with the intervening joint spared.

## HISTORICAL SURGICAL INTERVENTIONS

Antituberculous chemotherapy (ATT) is the mainstay of treatment of synovial or osteoarticular TB affecting the midfoot and hindfoot. Debridement, biopsy, synovectomy, or arthrodesis are common surgical adjuncts along with chemotherapy, and have specific indications. Some joints are disorganized at presentation. In these cases we can consider keeping the joint in distraction, especially after debridement, to supplement medical treatmentand retain some joint motion after disease resolution.

Surgical intervention helps to confirm definitive and early diagnosis, to achieve healing while maintaining function of the joint, preventing mid foot or hindfoot deformity in progressive disease, and for fusing joints when residual pain or deformity interfere with function or locomotion.

Synovectomy and debridement are traditional surgeries performed in tuberculosis of the foot, although the extent of synovium in foot joints is limited. Surgical synovectomy alone is not sufficient to achieve healing; nevertheless surgical intervention gives a chance for visualization of the damage to the joint surface. In early stages of the disease synovectomy alone maybe be helpful, while healing is achieved with chemotherapy. Typical watermelon seeds, large loose pieces of articular cartilage, can be removed and sent for histopathological study.

Stabilization of a joint that is extensively involved is of paramount importance; POP casts can be applied, but external fixation as an immobilizer or sometimes as a distraction aid may supplement the therapy.

The ankle joint is the largest and most mobile joint, and may constitute one third or more of the cases documented in the foot and ankle. Immobilization is imperative, and distraction in the early stages helps to maintain the joint surfaces and to preserve motion. Martini *et al.* [7] documented 54 cases involving the foot and ankle, but fusions were needed in 6 cases only; a subsequent publication by the same principal author [8] reported ankle TB in 36 out of the 520 musculoskeletal patients (6.9%); most presented with significant destruction, discharging sinuses and a 2/3 incidence of secondary pyogenic infection. These series, however, was published more than 30 years ago. Improved diagnostic methodology has reduced the number of late-presented cases in the 21^st^ century; nevertheless in the end stage of disease, fusion seems to be the acceptable option.

### Indications for Surgery

The indications of surgery in the early phases are principally for obtaining tissue diagnosis. Although fine needle aspiration would yield representative tissue in many cases due to the superficial nature of the foot structures, some deep-seated lesions, especially in the talus may need a formal biopsy [9]. Para-articular lesions threatening to involve a joint, especially in the midfoot maybe an indication for early debridement and biopsy to minimize spread across the whole midfoot; some chronic sinuses may need excision. Tubercular dactylitis has been frequently operated, but most commonly this is due to a mistaken diagnosis.

In the healed phase indications for surgery are primarily for pain relief, deformity correction or joint reconstruction [10]. The principal structure require reconstruction is the Tibio-talar joint [11], which often needs fusion, or the hind foot joints, which may also need to be stabilized occasionally.

## DEFINITIVE SURGICAL PROCEDURES FOR TB

Surgical interventions for TB need to be modified as for any infective condition.

1. Incisions have to include sinus tracts and standard incisions may not be feasible.
2. More than one incision may have to be taken and good planning is needed.
3. Thorough excision of infected tissues including bone needs to be done.
4. Infected granulation tissue has to be taken from the depth of infected structures for histopathology and culture.
5. Many cases have superimposed infection, therefore aerobic, anerobic, fungal and TB culture and sensitivity studies need to done. Recent introduction of GeneXpert test helps in detecting MDR cases within 24 to 48 hours and should be done if facilities are available.
6. Appropriate culture media should be kept ready in the operation theatre.
7. Soft tissue cover needs to be planned and obtained early.
8. The bone grafting/docking is generally done only after infection is under control.

A review of published reports on surgical interventions for foot and ankle TB is given in Table **1**.

## SURGICAL INTERVENTIONS FOR THE FOREFOOT

Dhillon *et al.* [1] published their initial experience with foot TB in 1993, and noted 7 of 22 cases involving the metatarsals or the Lisfranc’s joint. Only one of the 22, which involved the base of the metatarsal had to be fused, and one rocker bottom foot after healing was advised surgical fusion, which the patient refused due to limited disability. In purely bony disease of the foot and hands, children may get an expansile lesion; in foot it has been documented in more than one metatarsal, and the condition is termed “*spina ventosa*”. These lesions respond dramatically to medical management. The role of surgery is limited to biopsy and debridement, with appropriate anti TB medication that heals most cases if initiated early. Immobilization in correct position helps when the tarso-metatarsal joint is involved, as residual subluxations may lead to deformity. Healed cases in good position usually do well [3, 4].

An unusual presentation of forefoot disease maybe a cold abscess, which is rare in the foot, but can easily be seen on MRI (Fig. **4a**). This needs urgent decompression under cover of medical therapy.

## SURGICAL PROCEDURES FOR HINDFOOT AND MIDFOOT

A pubmed search with the keywords *“midfoot tuberculosis”* revealed only 13 hits, emphasizing the rarity of the disease in the foot overall, and the midfoot in particular. Many of these are published as isolated case reports limited to the talo-navicular or calcaneo-cuboid joints [2, 3, 12, 14], and the only documented procedure is biopsy or some degree of synovectomy.

The only indication for urgent debridement in the midfoot and hindfoot is para-articular bony disease threatening to invade an adjacent joint. This is true for calcaneus lesions as they can spread to the adjacent subtalar joint and cause limitation of motion and poor functional outcomes. In the midfoot all the joints are interconnected; articular disease, if left untreated, can spread across the whole midfoot and cause extensive damage. The presence of midfoot disease alone is not a primary indication for surgery; medical therapy will heal most cases, but immobilization in the proper position is mandatory during the early healing phase to minimize deformity progression and allow ambulation.

Dhillon *et al.* published 4 cases of osteomyelitis of the cuboid [2]; only one of these was operated due to a large osteolytic lesion with impending cuboid collapse. Being the most important structure of the lateral column, it is important to keep the cuboid bone from collapse, especially after curettage ordebridement. In this case a temporary external fixator was applied across the lateral column to keep the cuboid distracted till healing was observed. This is an unusual method of supporting the bone but seems to be biomechanically indicated. Curettage and primary bone grafting in the presence of active infection may not be a good option.

Hindfoot fusions as primary interventions for TB foot are not routinely needed; late stage disease in the underdeveloped world is still common, and some cases may present with deformed feet after healed TB of the hindfoot, and triple arthrodesis maybe a solution here. We have limited experience in this, and have noted that many healed cases of hindfoot disease do well despite arthritic changes across the joint (Figs. **5a**, **b**, **c**).

Arthroscopic procedures [12-15] have gained significant exposure with the advent of newer equipment and can be an excellent diagnostic aid as well as a biopsy tool. The published experience is limited as the keywords “*Foot + Tuberculosis, + Arthroscopy”* gave only 4 hits in pubmed, and most were related to the ankle or were done for another diagnosis. Lui and Stephen [16] published a case of ankle pain with a suggested diagnosis of Pigmented Villonodular Synovitis of the ankle and flexor hallucis longus tendon. Combined open and arthroscopic debridement revealed PVNS, but subsequent culture also grew tubercular bacilli. The importance of mycobacterium TB invading any pathological lesion was highlighted. It is important to consider TB as a differential diagnosis in all unusual foot inflammatory pathologies, to avoid missing the diagnosis of TB and getting pooroutcomes.

The subtalar joint seems to be a good area for arthroscopic debridement if it is needed, but there is no literature on this focused on TB. In many conditions subtalar arthroscopy has become an established technique, and it can be extended to cases of suspected TB also. Lui and Tong [17] have demonstrated that this is an easily applicable minimally invasive technique, with advantages of having less wound complications, faster rehabilitation and better cosmetic outcomes.

## RECONSTRUCTIVE PROCEDURES FOR ANKLE

Synovial biopsy is the most frequent procedure in the early stages of the disease; this would initiate anti tubercular medical treatment, which heals a large majority of the cases.

In the late stages of tuberculous arthritis, major reconstructive procedures are needed to reduce pain and improve gait; ankle fusion is preferred over total ankle replacement (TAR) as this has an unacceptably high incidence of complications as compared to ankle fusion in cases of tuberculosis [18].

Ankle arthrodesis is the end stage procedure for TB infection, ideally done for residual sequelae or for uncontrolled pain. Although the described techniques significantly differ in the surgical methodology and the choice of fixation, most rely on the concept of compression at the fusion site.

Regardless of the surgical technique chosen, the optimal postoperative position of the affected foot and ankle joint is the same. The foot should be externally rotated 20 to 30^0^ relative to the tibia [19], with the ankle joint in neutral flexion (0^0^), 5 to 10^0^ degrees of external rotation, and slight valgus (5^0^). This position provides the best extremity alignment and accommodation of hip and knee motion. Fusion of the ankle in plantar-flexion results in genu recurvatum when placing the foot flat on the floor and subsequent laxity of the medial collateral ligament of the knee, which develops from the externally rotated gait that patients adopt to avoid “rolling over” a plantar-flexed foot [19].

Arthrodesis in the presence of chronic active infection, inadequate soft tissue coverage, poor bone quality or the presence of established deformities represents a surgical challenge (Figs. **6a**-**g**).

### Open Ankle Arthrodesis

The technique of arthrodesis differs specifically as per the method of fixation used; this maybe determined by bone destruction and the stage of the disease, and the presence or absence of deformity. The soft tissue status or the presence of sinuses may modify the surgical incisions used.

The surgical steps common for TB ankle, regardless of the fixation modality used, include:

- Thorough soft tissue debridement
- Scraping of all periarticular osteolytic cavities
- Excision of remnants of articular cartilage
- Shaving of subchondral bone till fresh bleeding edges
- Compression and apposition of tibial and talar subchondral bone supplemented with autologous bone graft/allografts

Fixation under compression is essential for achieving bony union. The methods used may vary, and conventional implants are avoided in the presence of active infection.

Charnley utilized external fixation for compression arthrodesis, and this seems to be a particularly helpful method for achieving fusion without introducing implants in active infection [20]. Biomechanical studies showed that even though the Charnley device is able to produce sufficient compression forces, the single axis fixator does not produce rigid external fixation in all planes [21]. Rigidity of ankle fusion is improved by triangular compressive device that provides rigid external fixation in three planes, or hybrid frame circular external fixators. Compression across the arthrodesis site relies on an intact Achilles tendon functioning as a tension band. Patients are allowed to bear weight on the treated ankle during the first 8 weeks after surgery. After removal of the external fixator, patients are immobilized in a plaster walking-cast for an additional 4 weeks, and evidence of bony healing sought prior to allowing unrestricted ambulation.

In quiescent disease with residual pain or deformity, conventional fixation methods maybe used. These include

1. Anterior Plating, using a pre bent anterior plate fixed into the talar head and tibial diaphysis [22].
2. Posterior plating using a pre bent plate or a 95^0^ blade plate [23].
3. Transfibular exposure is a good option, where the distal 5- 10 cm of fibula is completely or partially excised and used as a strut bone graft. Fixation is done using two cancellous screws [24].
4. Screw fixation in various configuration can also be used as an isolated means of fixation, employing 2–3 cancellous screws [25]. The essential steps include correcting the mal-alignment, achieving raw bleeding tibio- talar surfaces and adequate compression with or without autologous bone graft.
5. Unique to TB or any other infective pathology is the need for local rotation/ microvascular flap with bone grafting, when significant amount of necrotic skin and soft tissue around the ankle joint has to be excised to achieve a healthy environment for subsequent arthrodesis [26]. The flap provides a thick vascular soft tissue cover for the bone graft and reduces incidence on non-union.

Many times, the combination of pantalar disease and osteoporotic bone precludes the use of standard internal fixation devices. Pantalar fusion is better obtained by intra-medullary device; when performed during active disease, the procedure should be done under appropriate chemotherapy cover to prevent disease spread or activation. Gavaskar and Chaudhary [27] have used a distal femoral nail in 7 patients of advanced grades of ankle TB arthritis; 5 had severe pain, and 2 could not walk without crutches. Sinuses were present in 3, gross articular destruction was documented in all. After arthroscopic synovial biopsy confirmed the diagnosis, joint debridement and complete synovial excision was performed, followed by multidrug chemotherapy for 12 months. They achieved fusion in all cases at a mean of 13months without relapse or implant failure.

Yoshida and co-authors [26] treated one middle aged and one elderly patient with debridement and arthrodesis. Both of these had been misdiagnosed either as septic arthritis or as PVNS. They performed an iliac osteo-cutaneous flap and a pan-talar arthrodesis was obtained. A vascularized iliac bone graft was performed and achieved successful triple fusion, but with a residual pseudoarthrosis of the ankle. Both cases healed from the disease, and had acceptable function. Vascularized bone graft offers the benefits of covering the bone defect, eradicating any sinuses, promoting bone union with drug delivery via the restored circulation.

## ROLE OF ARTHROSCOPY IN TB FOOT

Arthroscopy in ankle and hindfoot joints is helpful for biopsy and gives better view to determine the stage of the disease [13, 14]. Arthroscopic synovectomy and ankle fusion are becoming more frequent surgical interventions in tuberculosis (Figs. **7a**, **b**, **c**).

The traditional open approach for fusing the ankle has a few disadvantages; significant soft tissue stripping, inadequate exposure of the joint for the synovectomy, issues with post operative healing of wound are significant issues. A few authors thus suggest arthroscopic interventions for the ankle joint.

Classically arthroscopic ankle fusion is done for osteoarthritis or Rheumatoid arthritis of the ankle joint. Recent literature suggests successful treatment of Tuberculous arthritis of ankle joint by arthroscopy along with chemotherapy.

Tang and co authors [28] treated 10 patients of end-stage ankle tuberculosis, by arthroscopically assisted ankle fusion, and a half-ring external fixator. They used three approaches for arthroscopy, and were able to debride the joint adequately. They could start early weight bearing, and followed up their cases for a mean of 23 months. Their results were excellent, as all fusions healed; time to radiologic healing averaged 14.5 weeks, with very good outcome scores. External fixator provided good outcomes and they documented no recurrences. The key to good outcomes was good tibio-talar contact, which when stabilized under compression gives satisfactory outcomes. They concluded that when there is minimum deformity, arthroscopic ankle fusion has the advantage of minimum soft tissue stripping, clear preparation of the joint surface, radical debridement of the synoviumand fast post operative recovery.

## ARTHROSCOPIC ARTHRODESIS

This technique should be limited to patients with arthritic ankles with minimal deformity, because it is difficult to correct ankle deformity arthroscopically.

Advantages of arthroscopy are:

- Less post operative pain as there is less soft tissue stripping
- Preservation of blood supply of talus and tibia
- Preservation of malleoli
- Greater bone surface for fusion
- Less chance of malunion
- Can be converted to total ankle arthroplasty at a later stage

4.5-mm bur and curettes are used to denude the articular surfaces. After preparation, compression of the joint surfaces can be obtained with either internal or external fixation. Preferably, two cannulated screws are placed across the tibia into the talus. The first screw runs from the lateral aspect of the tibia into the neck of the talus. The second screw runs from the medial malleolus into the lateral aspect of the talus.

## CONCLUSION

Foot and ankle TB is uncommon; the ideal management is by medical therapy, especially when diagnosed early and instituted in appropriate doses; Bone destruction in the weight bearing joints can potentially cause deformity and interfere with ambulation. Historical surgical interventions range from synovectomy and debridement, which aids diagnosis, to distraction in an attempt to maintain joint space during healing, to fusions for painful disorganized joints. External fixators can help both in distraction of joints as well as for compressing joints during fusion. Surgical planning should take into account of sinuses and scars, as well as the need to excise unhealthy skin; the need for flaps and plastic surgery should be kept in mind. Arthroscopy has proven to be a valuable adjunct both for diagnosis and biopsy, as well as a tool for arthroscopic aided fusions with the potential for easier subsequent interventions.

## Figures and Tables

**Fig. (1a):**
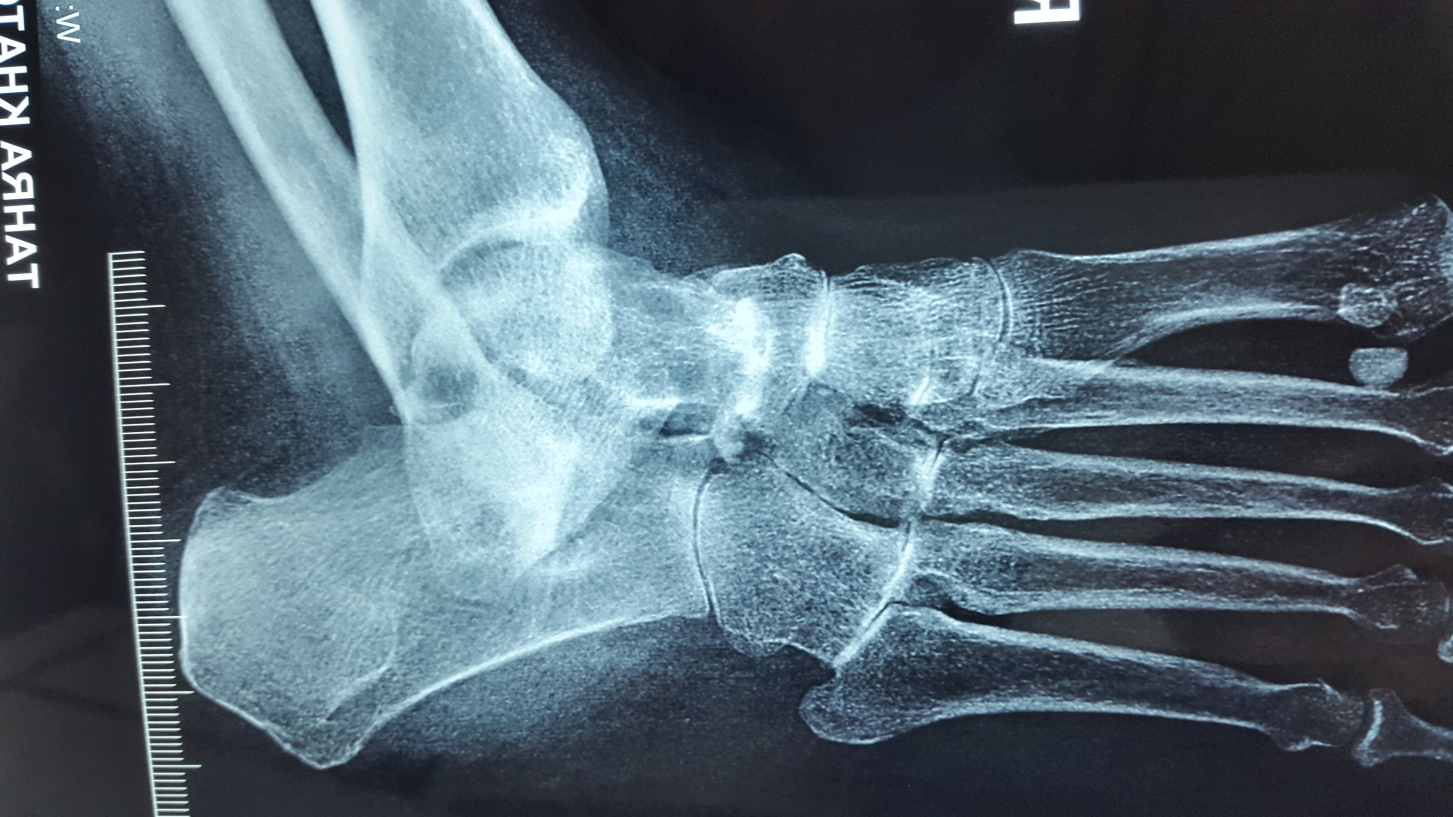
Midfoot tubercular arthritis which presents as a rheumatoid type lesion, with gross osteopenia.

**Fig. (1b):**
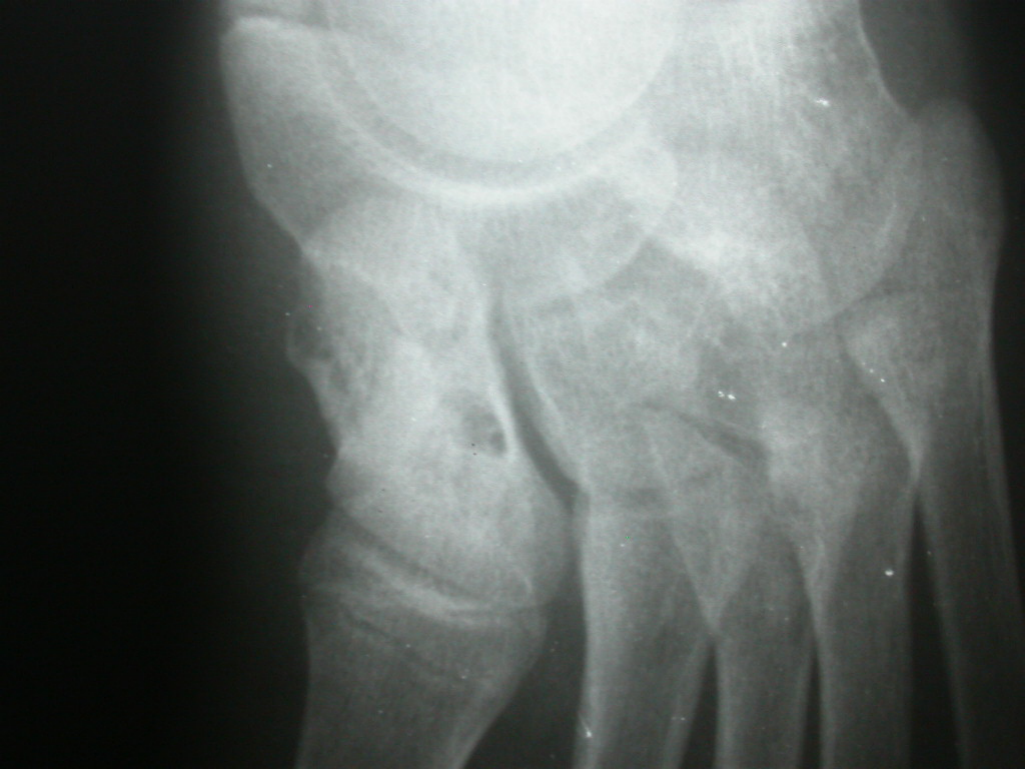
Irregular osteolytic lesion on medial cuneiform.

**Fig. (1c):**
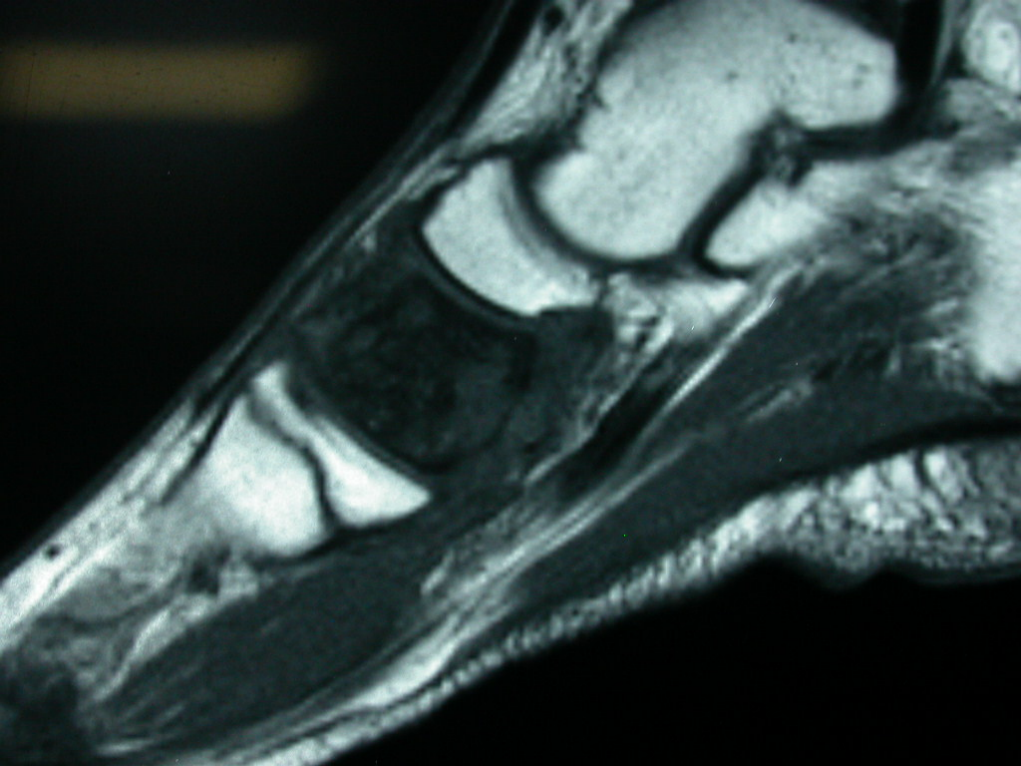
MRI of 1b, showing signal changes in whole of medial cunieform.

**Fig. (2a):**
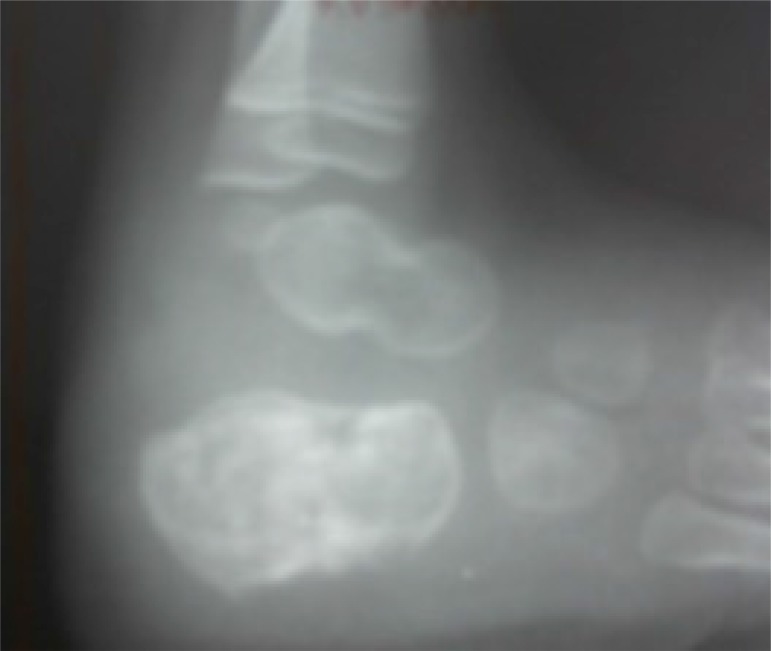
Expansile osteolytic lesion of calcaneus in a child.

**Fig. (2b):**
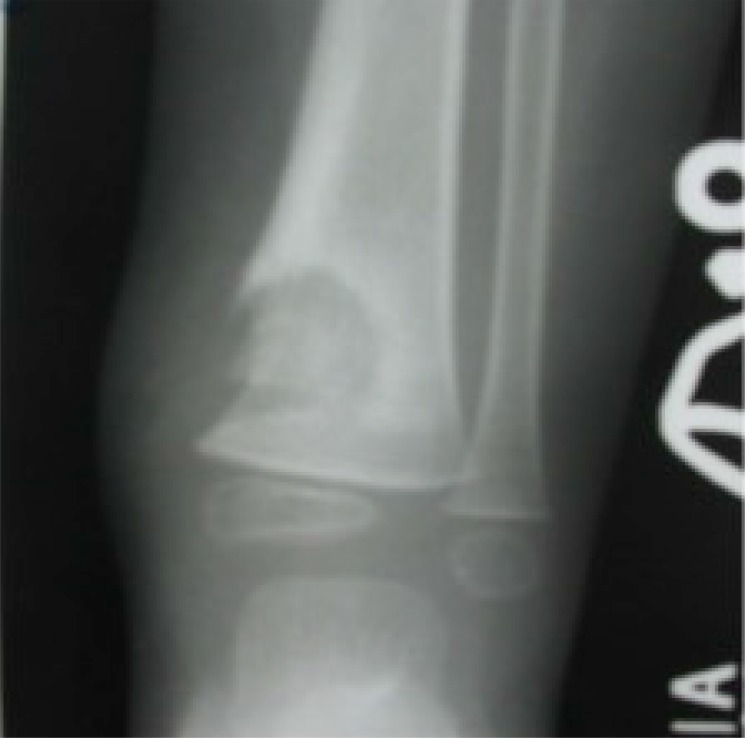
Distal tibial osteolytic lesion with flaky sequestrum.

**Fig. (3a):**
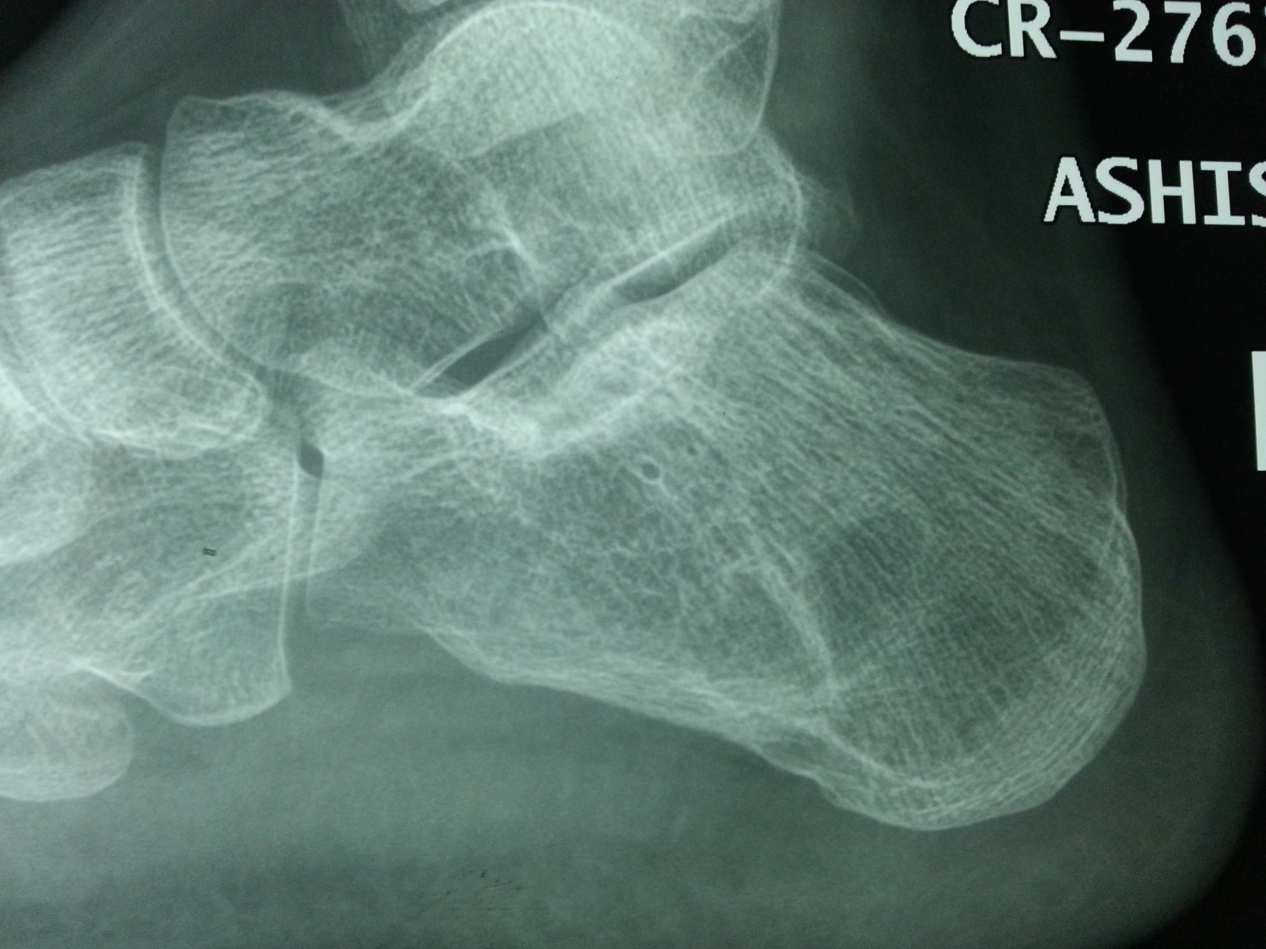
Lateral view of calcaneus with suspected osteolytic lesion.

**Fig. (3b):**
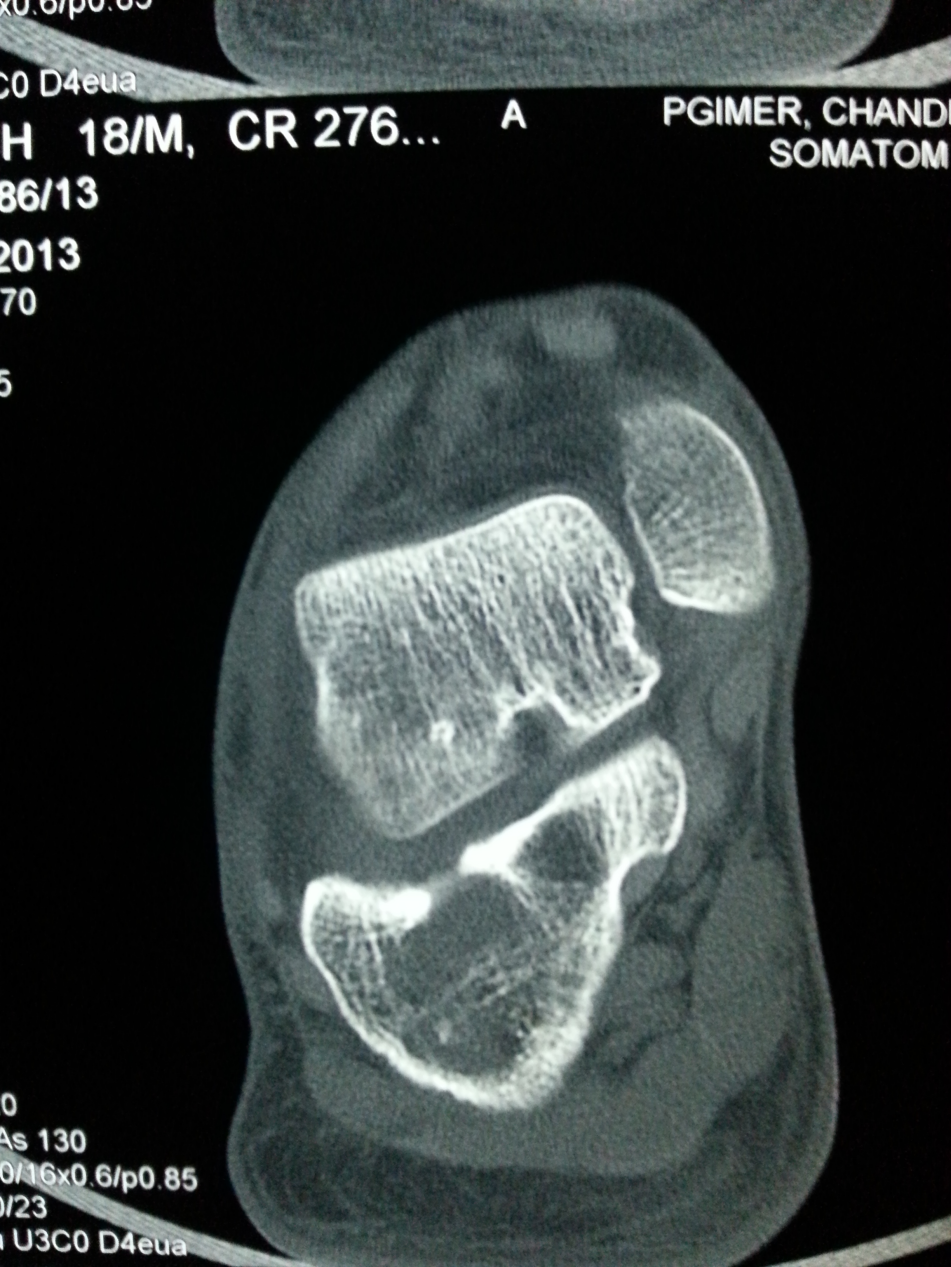
CT scan showing extensile destructive lesion in same case.

**Fig. (3c):**
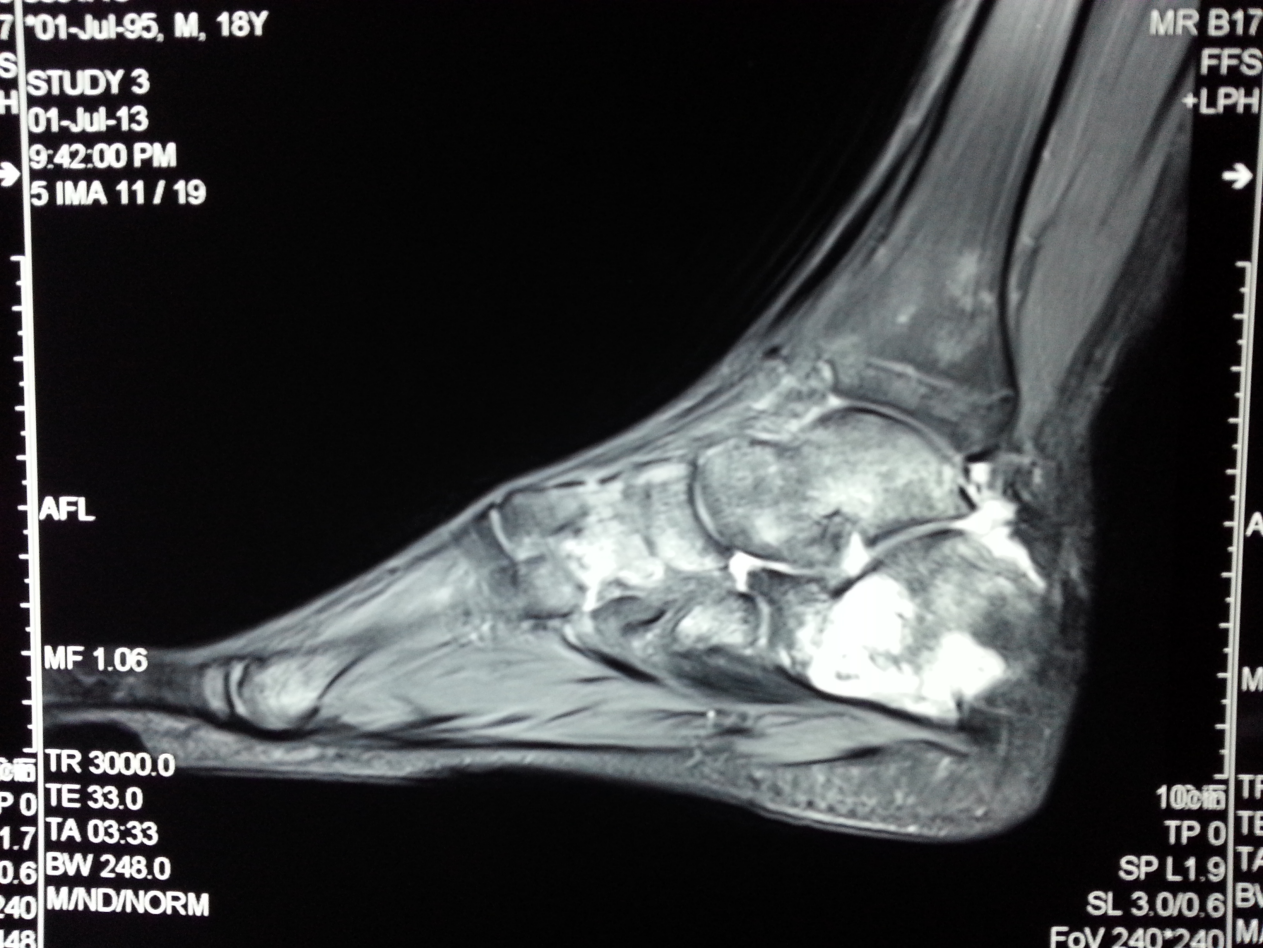
MRI of the same case showing fluid in subtalar joint and signal changes in most hindfoot bones.

**Fig. (4a):**
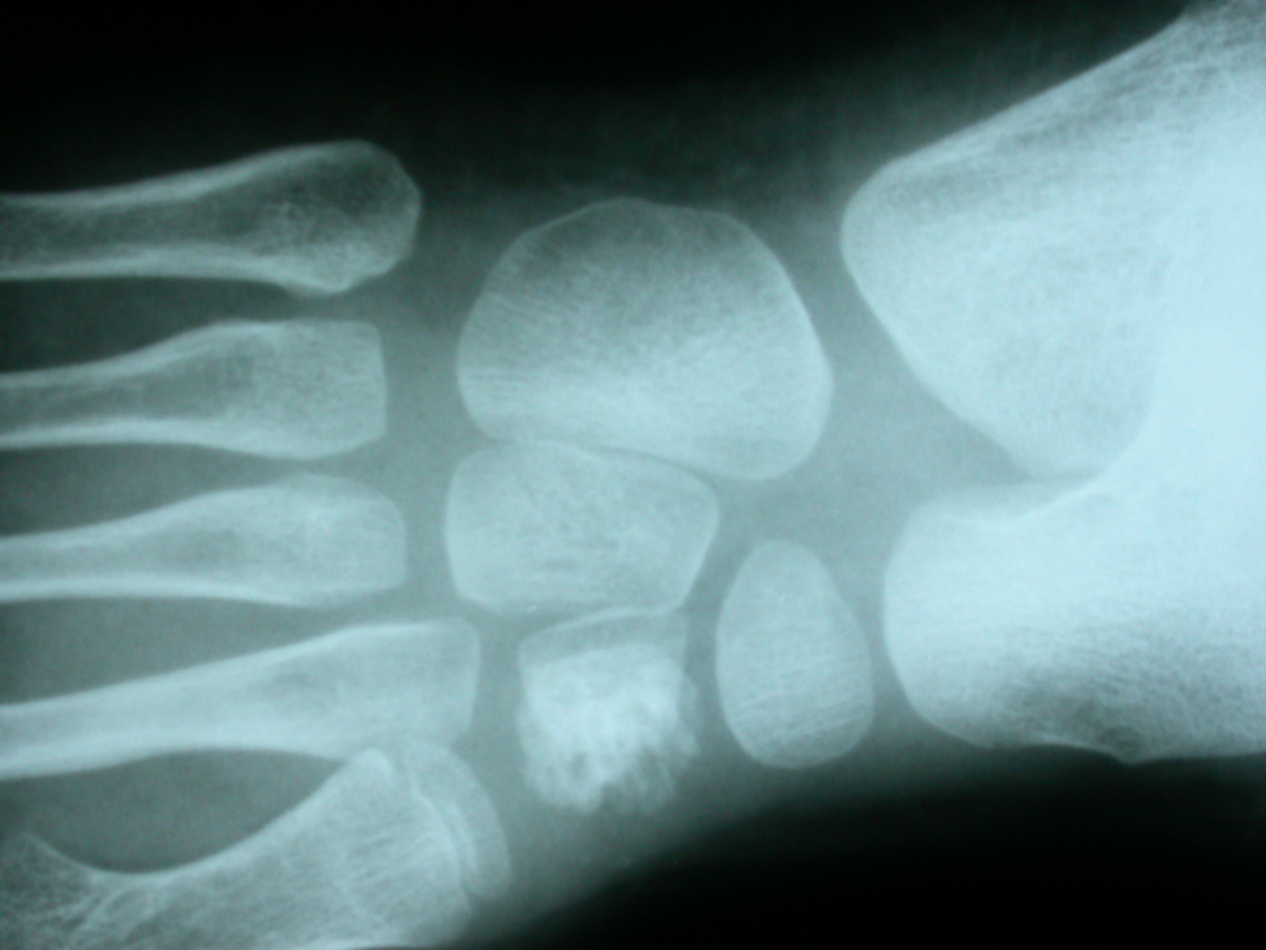
Painful, swollen foot of a child.

**Fig. (4b):**
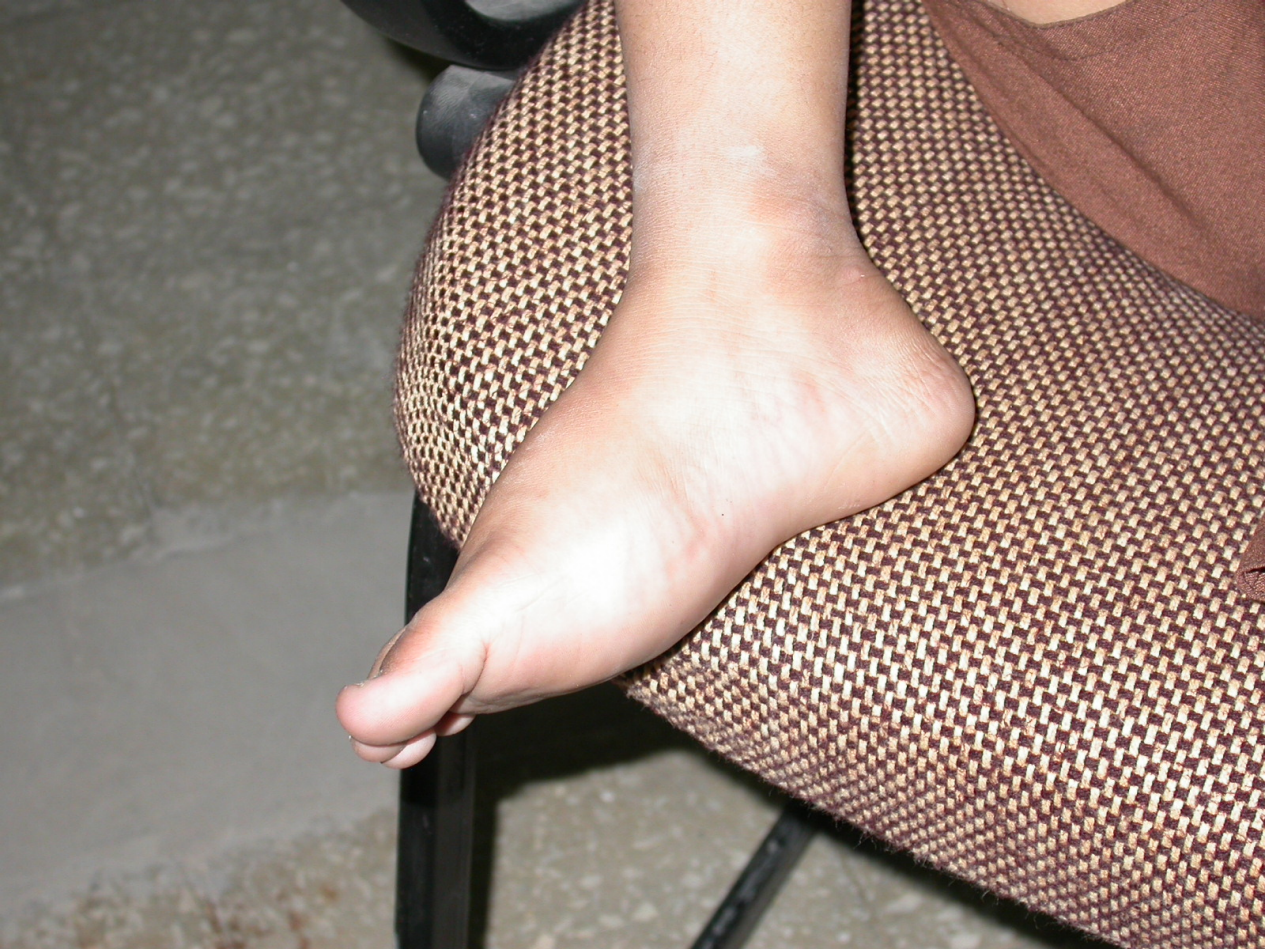
X-Ray (AP view) showing irregular destruction of medial cuneiform.

**Fig. (4c):**
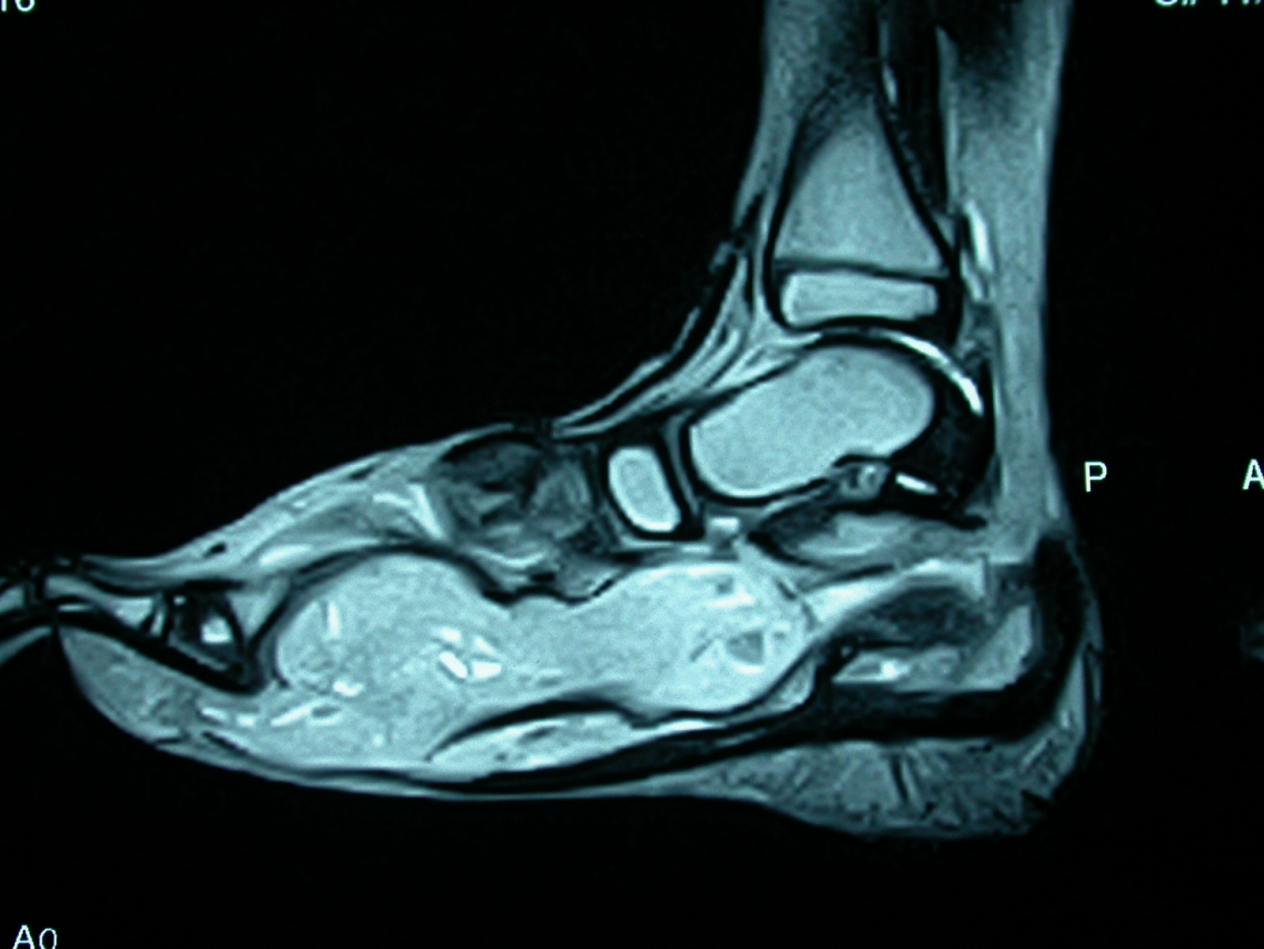
MRI showing large cold abscess on the planter surface.

**Fig. (5a):**
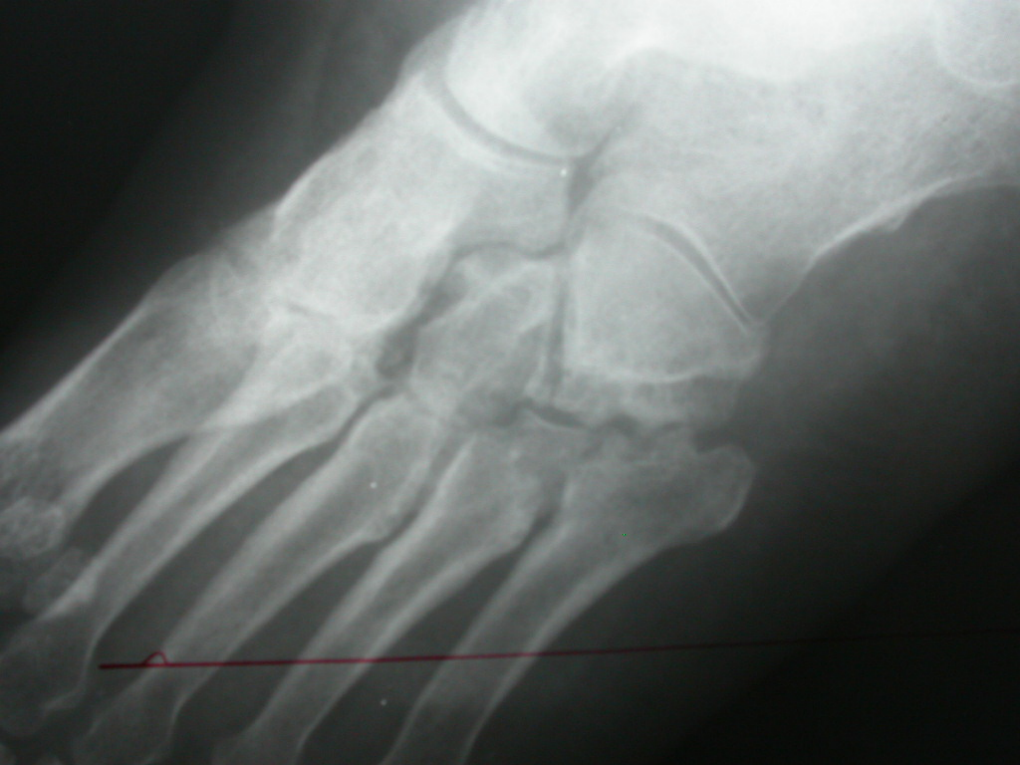
Healed tarso-metatarsal TB with painless lisfrancs joint at 9 years.

**Fig. (5b):**
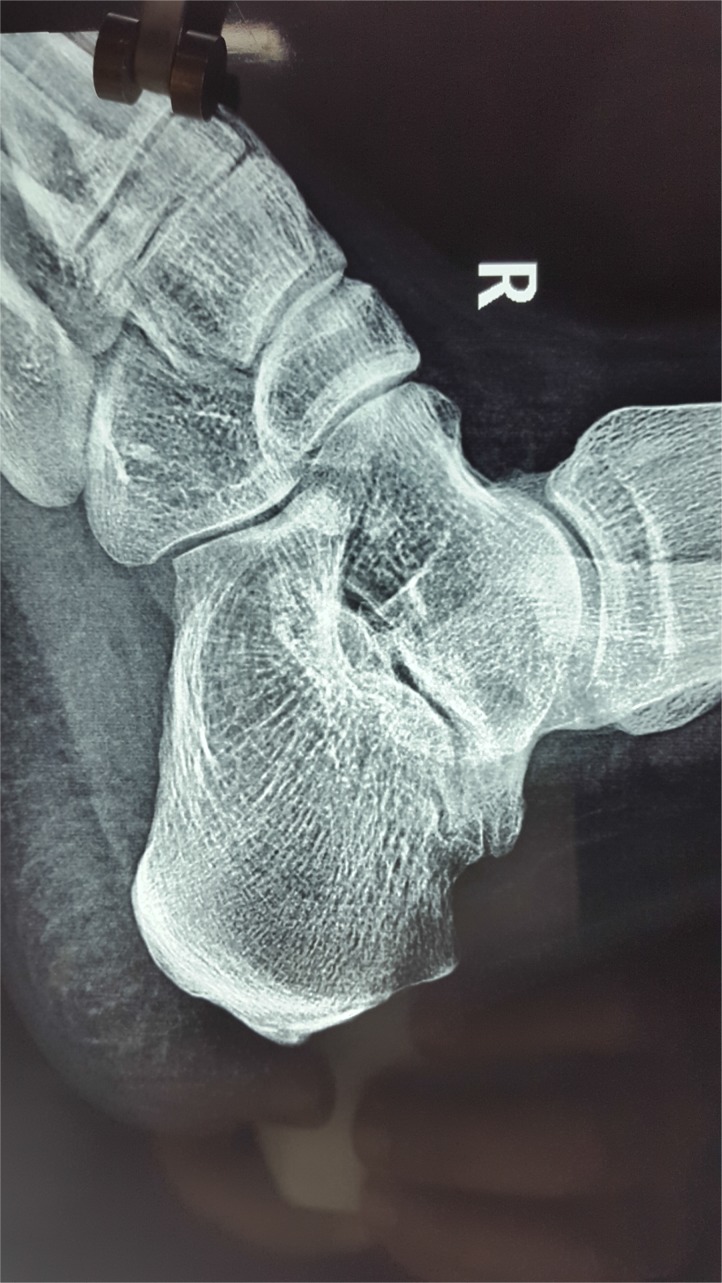
21 years follow up of healed sublatalar joint disease (case reported previously in 1993, *Dhillon *et al.* [**1*]) with painless activities.

**Fig. (5c):**
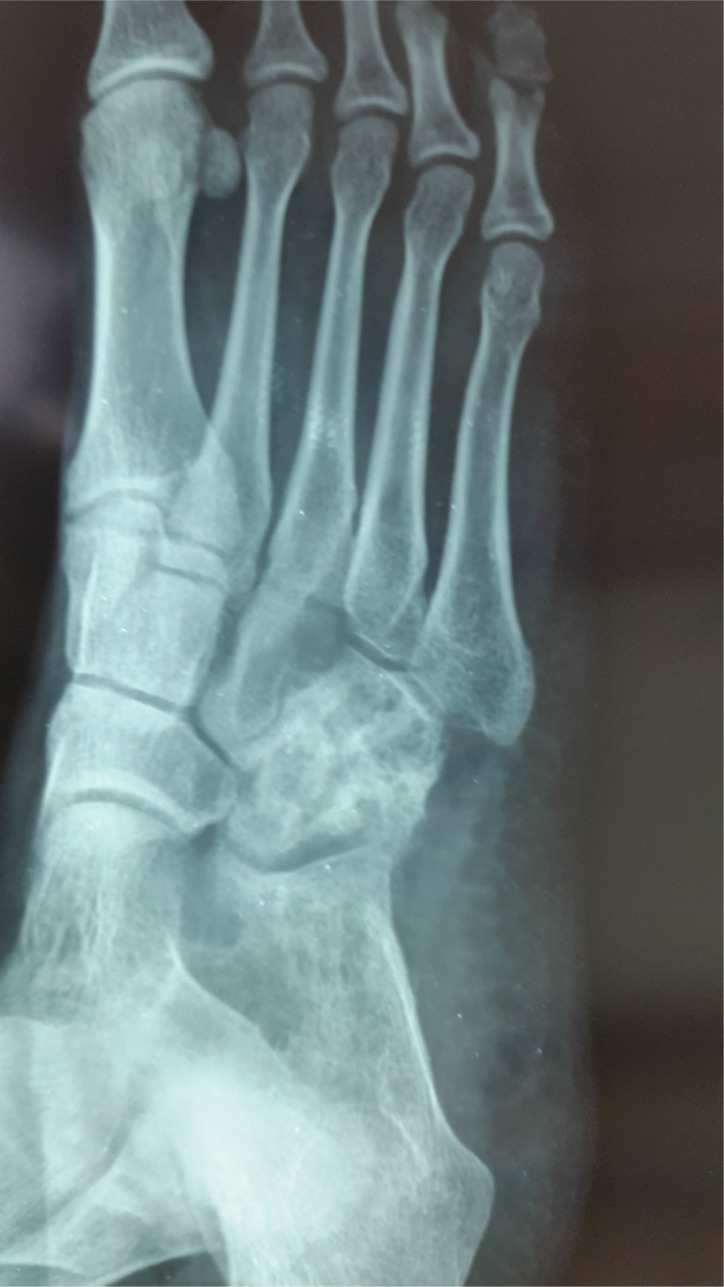
Healing response in Cuboid osteolytic TB within 6 months of ATT.

**Fig. (6a):**
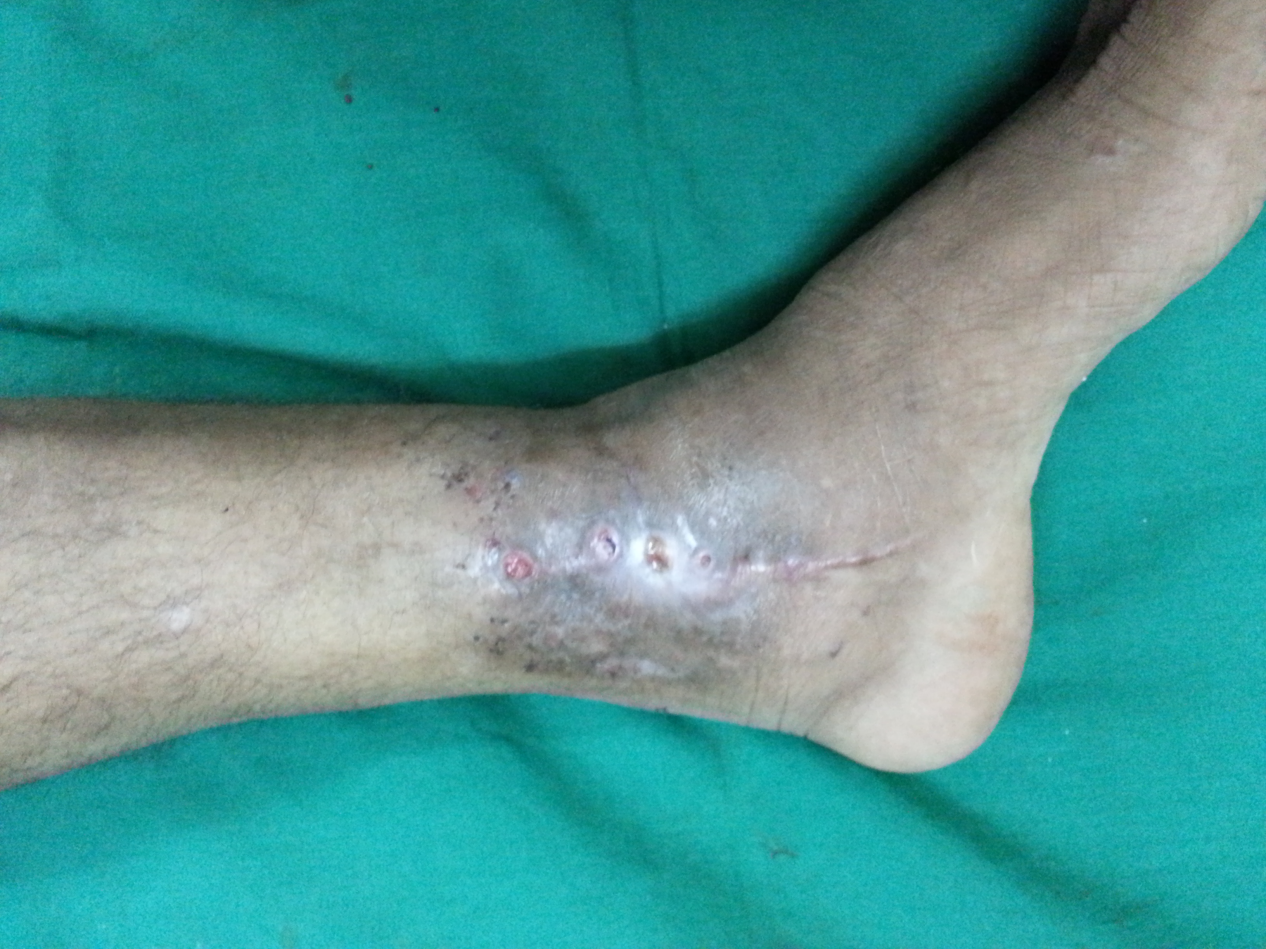
Discoloured skin and discharging sinus of TB ankle with irregular treatment and secondary infection.

**Fig. (6b):**
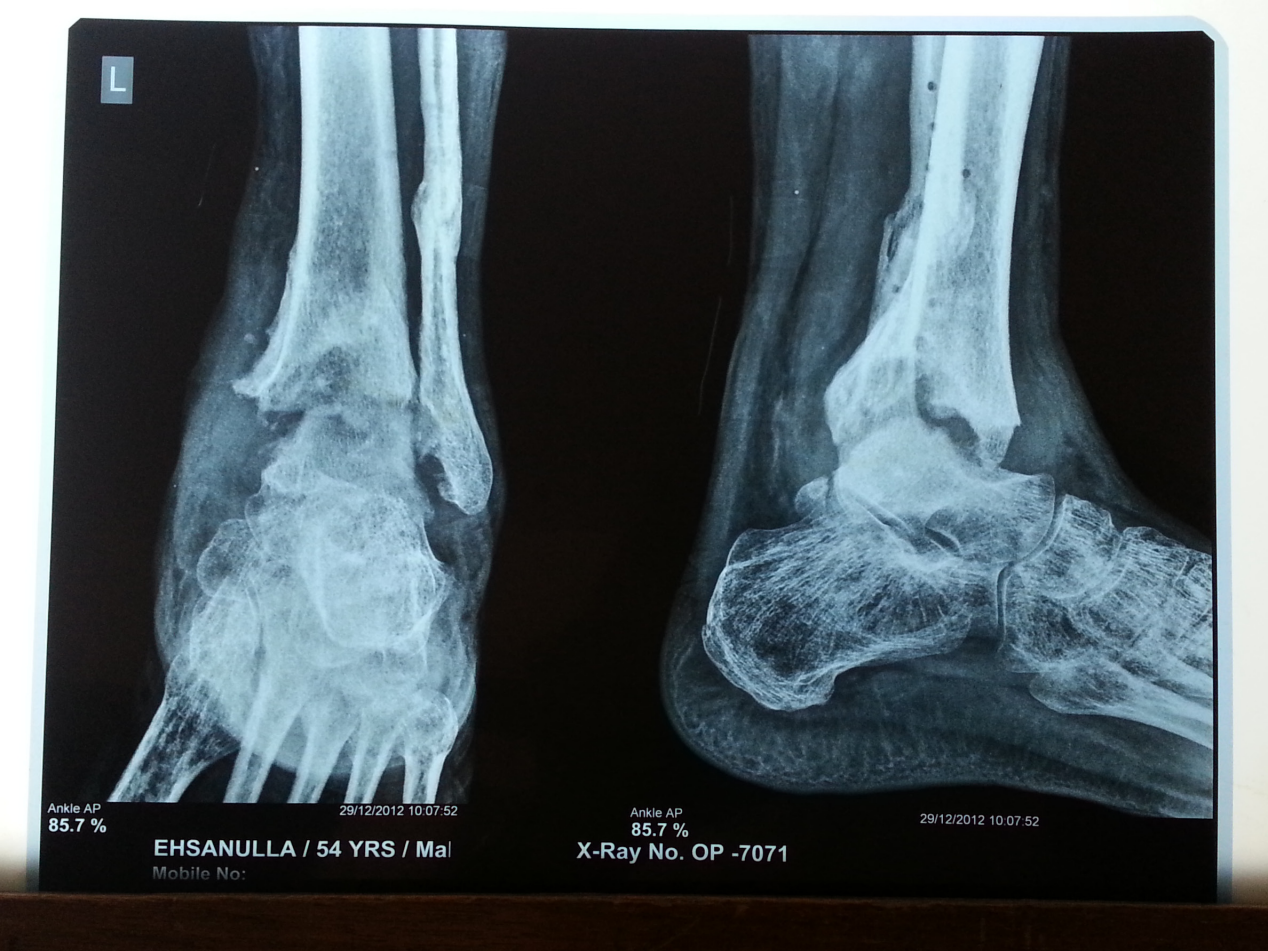
AP and Lat view of Xray showing joint destruction.

**Fig. (6c):**
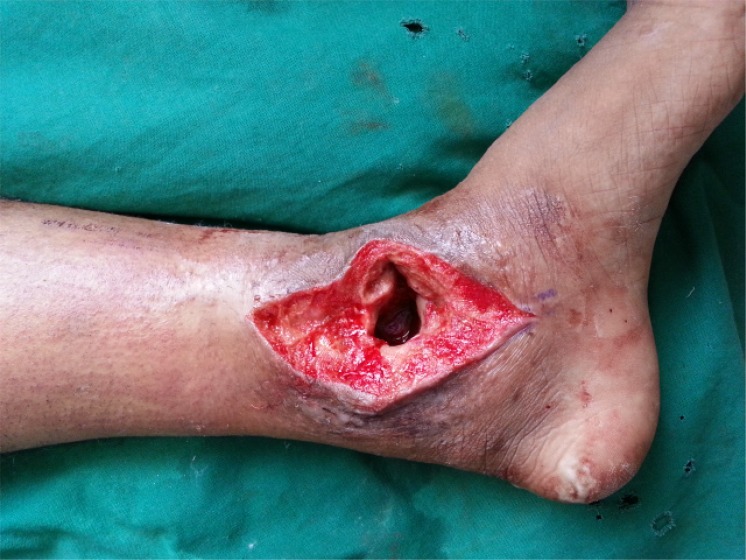
After debridement of sinus and unhealthy tissue.

**Fig. (6d):**
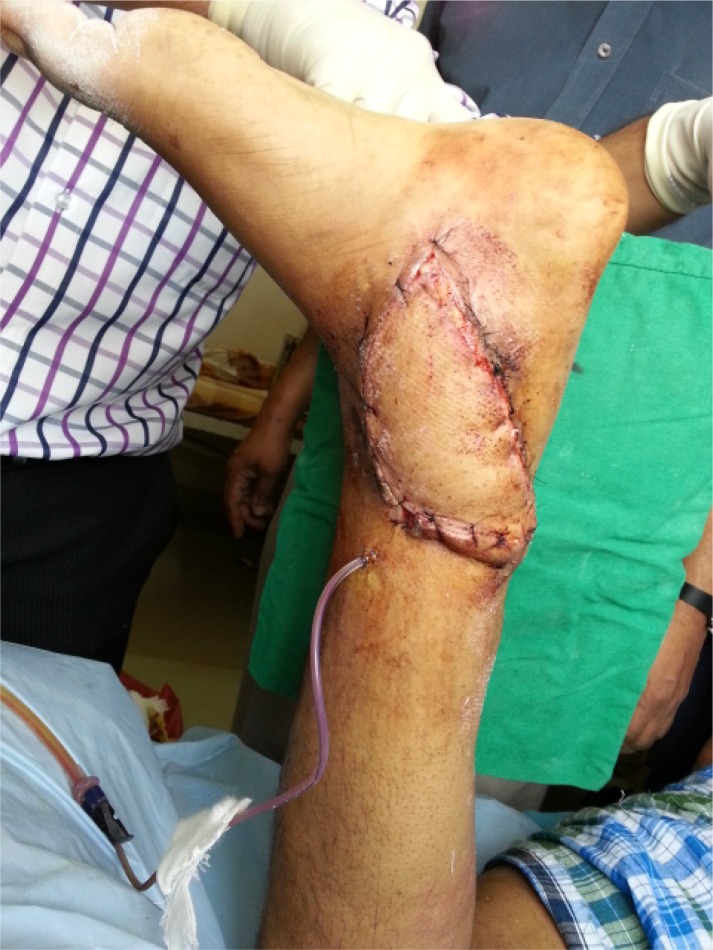
Soft tissue cover by flap and fixation by external fixator; fibula was used as supplemental bone graft.

**Fig. (6e):**
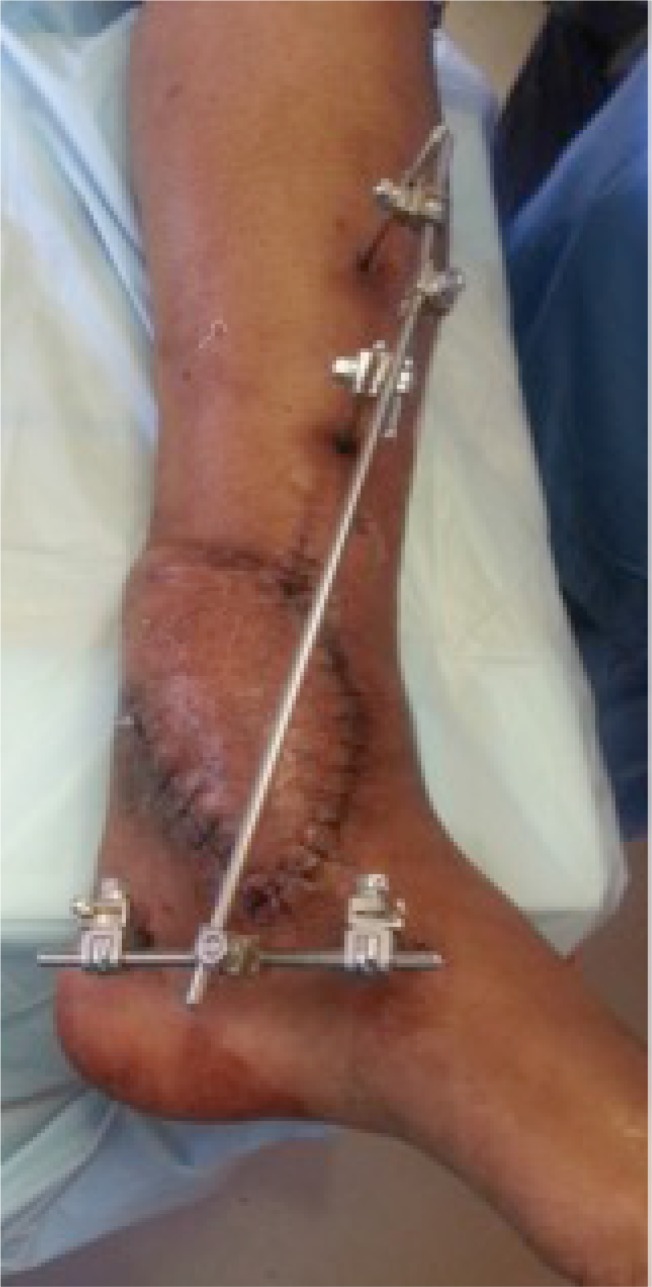
Healed flap.

**Fig. (6f):**
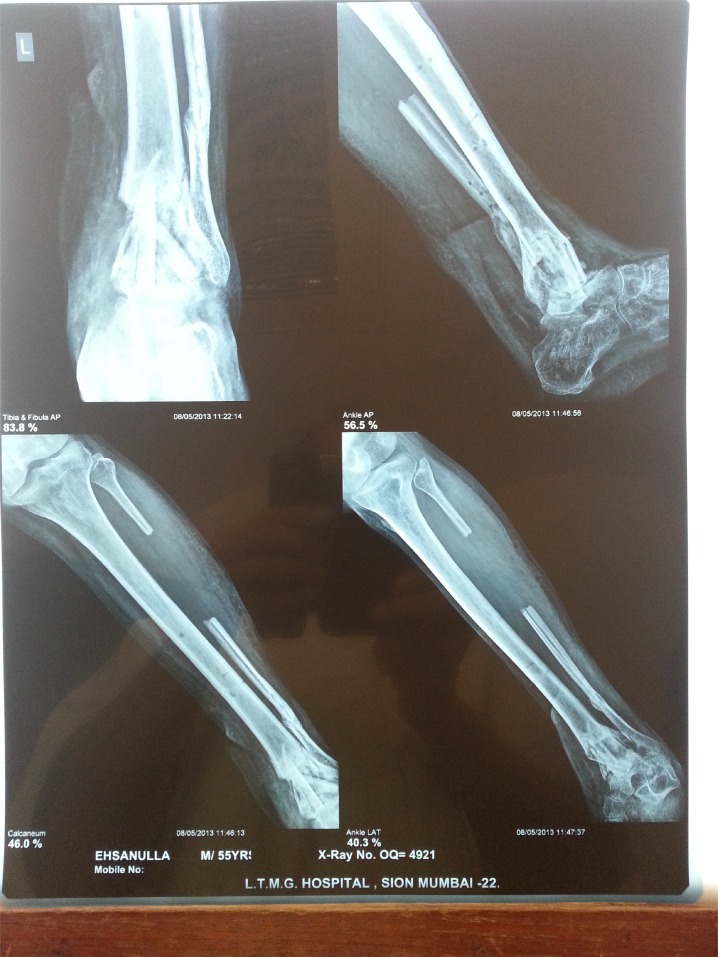
Follow up x-rays.

**Fig. (6g):**
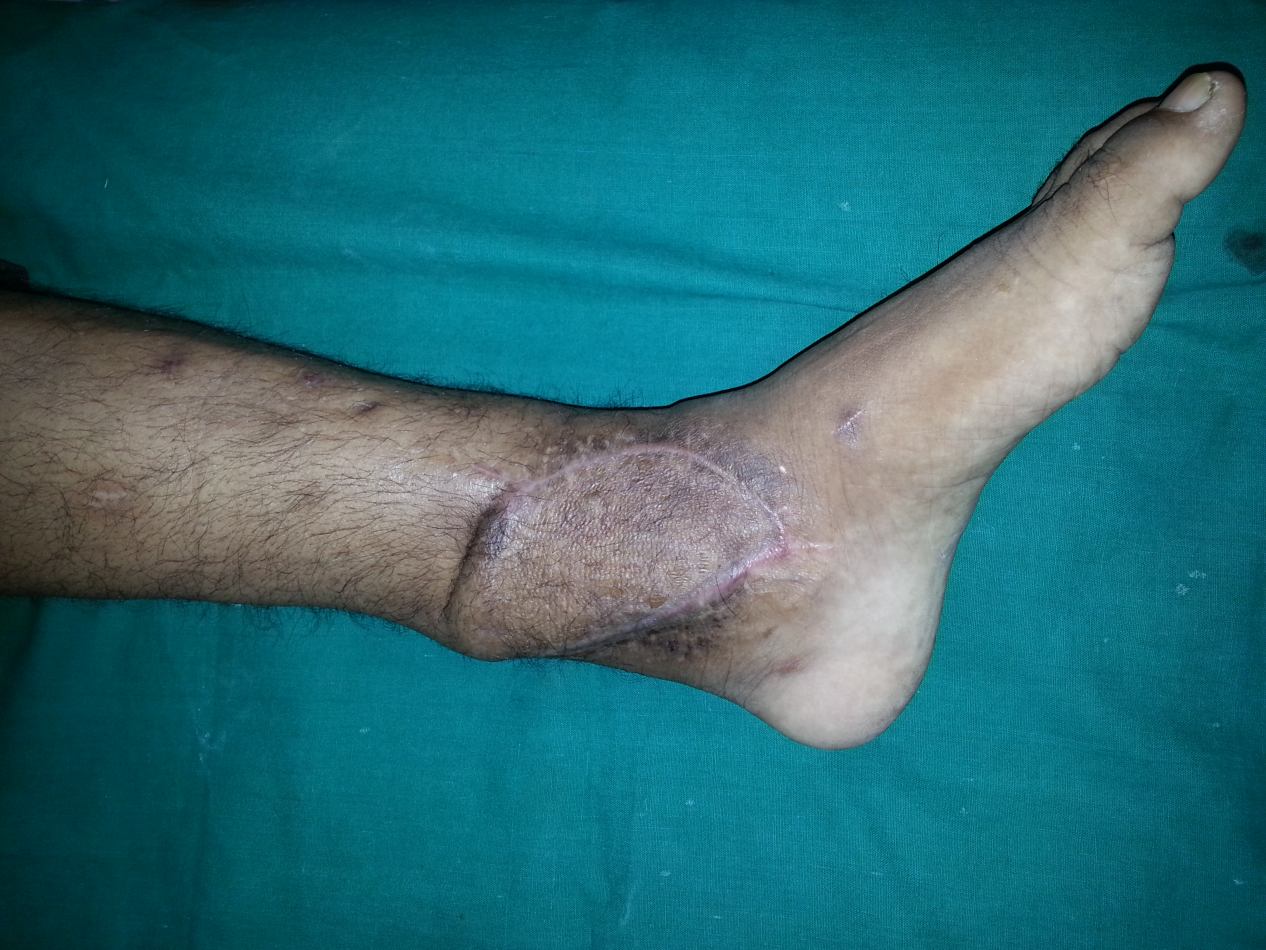
Healed wound with flap in situ.

**Fig. (7a):**
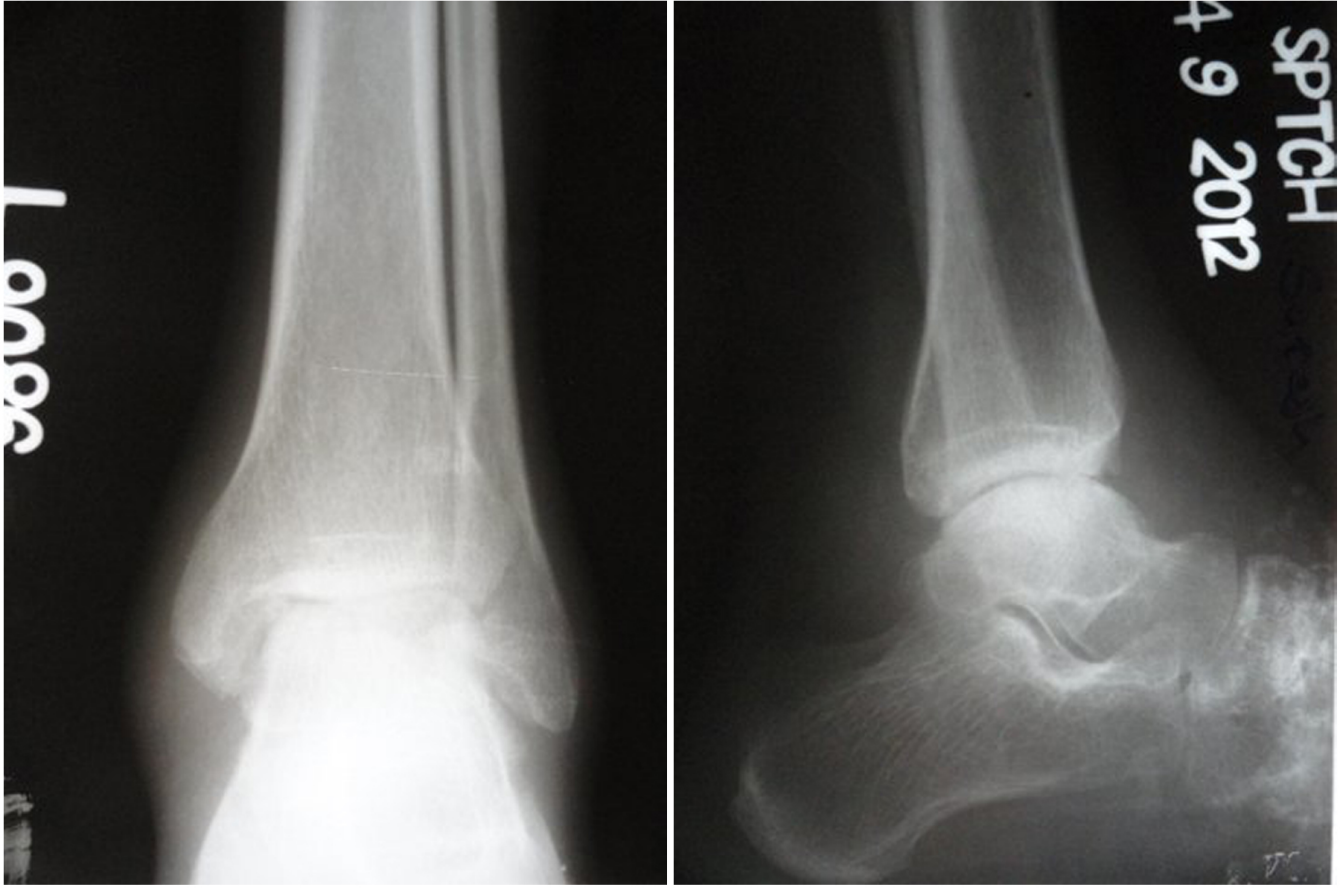
AP and Lateral view of TB ankle with painful gait after medical treatment.

**Fig. (7b):**
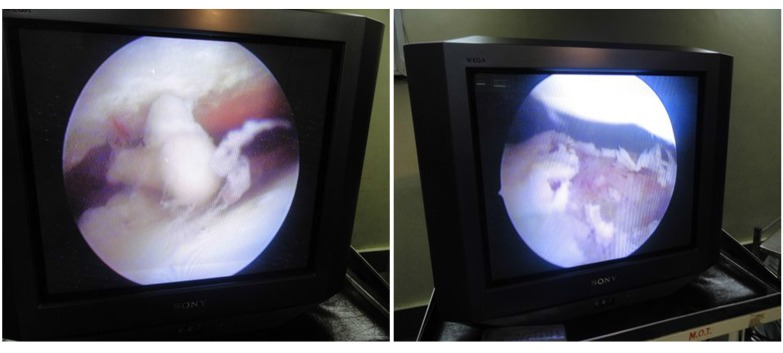
Arthrosocopic view of the joint.

**Fig. (7c):**
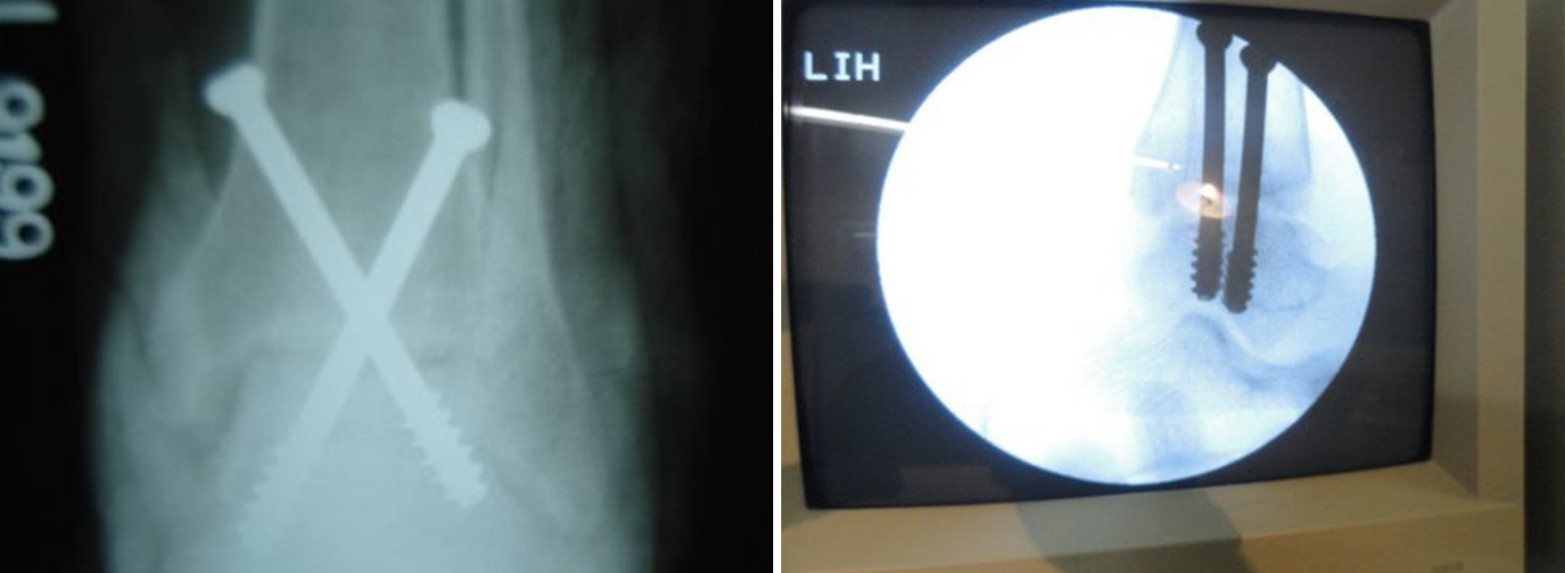
After arthroscopic arthrodesis and screw fixation.

**Table 1:** Literature review of Surgical procedures for Foot and Ankle TB.

**Author/year**	**Foot cases**	**Ankle cases**	**Synovectomy/ debridement**	**Fusion**	**Outcome**	**Comments**
Chen *et al.* 2013	nil	29	15	Ankle 11 Subtalar 3 Talonav 1 >2 peritalar joints 4	61 procedures on 29 cases. 8 mixed infections.	Late talar collapse is a problem.
Tang *et al.* 2007	nil	10	Arthroscopic synovectomy and debridement 10	10 ankle fusion with half ring external fixater.	Mean radiographic fusion 14.5 weeks.	No recurrence
Lin *et al.* 2009	nil	2	Arthroscopic synovectomy and debridement 2	Nil	Healed disease, good function, retained ankle motion.	High awareness in immune compromised patients.
Gavaskar & Chaudhary 2009	nil	7	Arthroscopic synovial biopsy 6	Fusion with IM nail 7	Healed disease, re mineralization <1 year in all	Reliable method, allows early mobilization.
Dhillon & Nagi 2002	61	13	Calcaneus curettage 3, Distraction of cuboid 1	Triple arthrodesis 3, Talonavicular fusion 1	Bilateral 1 1 death due to hepatic involvement Most resolved with medical ATT	Limited need for surgical intervention if diagnosed aerly.
Martini *et al.* 1984	26	32		Ankle arthrodesis 5 Triple fusion 1	All responded to ATT one relapsed after 2 years	Arthrodesis seldom needed and should be done for pain or deformity.
